# Relationship between Cervicocephalic Kinesthetic Sensibility Measured during Dynamic Unpredictable Head Movements and Eye Movement Control or Postural Balance in Neck Pain Patients

**DOI:** 10.3390/ijerph19148405

**Published:** 2022-07-09

**Authors:** Ziva Majcen Rosker, Miha Vodicar, Eythor Kristjansson

**Affiliations:** 1 Faculty of Sport, University of Ljubljana, 1000 Ljubljana, Slovenia; 2 Department of Orthopaedic Surgery, University Medical Centre Ljubljana, 1000 Ljubljana, Slovenia; miha.vodicar@kclj.si; 3 Orthopaedics, Medical Faculty, University of Ljubljana, 1000 Ljubljana, Slovenia; 4 Landspitali University Hospital, 101 Reykjavik, Iceland; eythork@simnet.is

**Keywords:** sensorimotor functions, neck pain patients, balance, oculomotor control, proprioception

## Abstract

Cervical afferent input is believed to affect postural balance and oculomotor control in neck pain patients, but its relationship to cervicocephalic kinesthesia, describing movement sense, has not yet been studied. The aim of this study was to analyze the relationship of two aspects of cervicocephalic kinesthesia to postural balance and oculomotor control in neck torsion positions. Forty-three idiopathic neck pain patients referred from orthopedic outpatient clinics and forty-two asymptomatic controls were enrolled in the study. A force plate was used to measure center-of-pressure movements during parallel stances under neutral and neck torsion maneuvers. Video-oculography was used to assess eye movements during smooth pursuit neck torsion test (SPNTT), while kinesthetic awareness was measured using the Butterfly test and head-to-neutral relocation test. Multiple regression was used to describe relationships between tests. Body sway in the anterior–posterior direction was related to Butterfly parameters but less to the head-to-neutral test. A medium relationship between Butterfly parameters and gain during SPNTT, with less SPNT-difference, was observed, but not for the head-to-neutral test. It can be concluded that specific aspect of neck kinesthetic functions (i.e., movement sense) importantly contributes towards oculomotor and balance control, which is more evident under neck torsion positions in neck pain patients, but is less pronounced in asymptomatic individuals.

## 1. Introduction

Neck pain disorders have been identified as one of the most challenging chronic medical conditions with an exponentially increasing number of cases in years lived with disability [1]. While over the last three decades one of the most commonly reported outcome measures in clinical and research practice was pain, it is now clear that the elimination of pain itself does not prevent reoccurrence and chronicity [2]. Possible reasons for reoccurrence and chronicity might be disturbances in the sensorimotor control system commonly observed in patients with neck pain disorders [3].

Sensorimotor disturbances affect multiple functional subsystems of which altered postural balance [4], eye movement control [5,6], kinaesthesia [7] and others are commonly identified in patients with neck pain disorders. A possible reason for sensorimotor disturbances in neck pain patients is suggested to be due to mismatch of sensory information derived from the cervical spine, visual and vestibular systems, which are neurophysiologically interconnected at the level of the brainstem [8]. The present mismatch is reflected as an altered ability of the accurate perception of head, neck and whole body position and movement in space (i.e., kinesthetic awareness) [9,10].

Alterations in cervicocephalic kinesthetic sensibility have been identified in previous studies investigating neck pain patients, confirming disturbances in position sense [11]. As position sense measures only one aspect of proprioception [12] applying more complex tests measuring movement sense and movement control during dynamically changing position is of importance [13]. Sensory information from different structures (i.e., muscle spindles, Ruffini corpuscles, Pacinian corpuscles and others) are integrated in movement sense [12,14]. This is of importance as idiopathic neck pain patients may suffer from multiple structural and functional impairments [7,15,16]. In addition, the pathological ingrowth of certain proprioceptors (Ruffini corpuscles) into cervical structures (i.e., intervertebral discs) can produce false sensory information, consequently influencing coordination of alpha and gamma motor neuron activity [16]. This is important for muscle spindle function which can negatively influence head and neck movement sense and consequently decrease their movement control [9,10].

Cervical afferent input is believed to importantly contribute to postural balance [17,18] and eye movement control [19]. Abnormal cervical afferent input is suggested to cause disturbances in the cervico-collic and cervico-ocular reflexes, consequently negatively influencing eye movement control, especially in neck torsion maneuvers [20,21]. Same trends of poorer performance during neck torsion maneuvers have been proposed for postural balance tasks [22] but their relationship to afferent input reflected in dynamic cervicocephalic kinesthesia has not yet been measured in neck pain patients. Although the literature implies that the aforementioned aspects of sensorimotor control present different functional characteristics [23], some evidence exists that suggests they are interrelated [3]. Recent articles describe existing correlations between postural balance, eye movement control and dynamic cervicocephalic kinesthesia [19,24]. However, this relationship was only studied in healthy athletes where habitual postural adaptations were proposed as possible reason for asymmetrical tonic activity of neck muscles, causing sensory mismatch. As described above, sensory mismatch is suggested as one of the main reasons for sensorimotor impairments in patients with neck pain disorders. Therefore, the aim of this study was to analyze the relationship of two aspects of cervicocephalic kinesthesia (head-to-neutral relocation test and Butterfly test) with postural balance and oculomotor control, in neck torsion positions in both neck pain patients and asymptomatic individuals.

## 2. Methods

### 2.1. Participants

Asymptomatic controls and idiopathic chronic neck pain patients were referred to the study. Patients were recruited from the orthopedic clinic of the national University medical center, and three private orthopedic outpatient clinics and were assessed for suitability via a telephone interview prior to participation by an experienced physiotherapist. In addition, healthy subjects were recruited for the study and were randomly selected between university staff, doctoral students and their friends. To be enrolled, all participants had to present with the following inclusion criteria: more than 50° of cervical rotation to each side, age between 18 and 55 years, and the group of idiopathic neck pain patients had to present with a minimum of four for pain in the neck on a visual analogue scale. Participants were excluded if they reported: previous traumatic injuries to the head or neck, shoulder, upper or lower extremity pain within the last two years, any neurological or vestibular disorders, type II diabetes, diagnosed psychiatric disorders. Furthermore, all participants were required to refrain from taking medication or alcohol 30 h prior to the study. Participants had to read and sign a consent form and were free to withdraw at any time. The study was approved by the national medical ethics committee (number: 0120-47/2020/6) and was performed in accordance with the declaration of Helsinki and its later additions.

### 2.2. Measurement Procedures

Patients were required to mark pain intensity on a visual analogue scale and underwent magnetic resonance imaging assessment prior to an initial screening at the orthopedic outpatient clinics. This information was used to describe the extent and variability of cervical spine structural impairments. Tests of body sway, smooth pursuit neck torsion test (SPNTT), neck kinesthesia using the dynamic kinesthetic awareness test (Butterfly test) and position sense (head-to-neutral) were performed by an experienced physiotherapist.

Reliable and valid measures of postural balance [25] were assessed in randomly ordered postural tasks; upright quiet parallel stance with feet positioned parallel at the hips` width parallel stance with neck torsioned to 45° to the left and right. During all stances, participants were instructed to place their hands on the hips and to keep their knees straight. All balance tasks were performed standing on a force plate measuring the center of pressure (CoP) movement (9260AA, Kistler Instruments AG, Winterthur, Switzerland). During testing, participants had to maintain their vision on a target positioned at a 2-metwe distance. Each stance was repeated three times for 30 s separated by 60 s rest intervals.

A reliable and valid SPNTT protocol [5,6,26] was conducted as described in the study by Majcen Rosker et al. [6]. Tracking of a horizontally moving target was performed at three different neck positions: facing forward (the trunk and head were in a neutral position), right neck torsion at 45° and left neck torsion at 45°. Eye movements were measured using infrared video-oculography (Pro Glasses 2, Tobii, Danderyd, Sweden) at a sampling rate of 100 Hz [27,28,29].

Participants were sitting on a custom-made rotatable chair and were required to track 10 cycles of cyclic sinusoidal target movements with their eyes for each condition, followed by 60 s rest interval. A horizontally moving target was projected (Optoma ML1050ST LED Projector, Fremont, CA, USA) on a white screen 150 cm away at eye level. Participants were tested at a target movement profile using 40° of target movement amplitude with a velocity of 30°/s at all three neck positions in a random order.

Valid measurements of cervicocephalic kinesthetic awareness were taken using the Butterfly test (formally the Fly test) [13,30] and head-to-neutral relocation test [31,32,33]. During the Butterfly test, participants were instructed to accurately follow a dynamic unpredictable target with their neck. Cervicocephalic kinesthesia was measured using an inertial measurement unit (NeckSmart, NeckCare Holding ehf., Reykjavik, Iceland). Two repetitions of three different movement paths of increasing difficulty (easy, medium and difficult) were used. Target movement path characteristics and test duration were predefined by the NeckCare software (NeckSmart, NeckCare Holding ehf., Reykjavik, Iceland).

A head-to-neutral relocation test was used to measure error in the position sense of the cervical spine. Prior to the initiation of each measurement trial, participants had to position their head and neck into a self-selected neutral position, serving them as a reference position. While blindfolded, each participant performed three repetitions of slow head movements to both rotations, flexion, or extension and back to the predetermined neutral position. The head-to-neutral relocation test was performed using the same inertial measurement unit and software as described in the Butterfly test.

### 2.3. Data Analysis

Signals derived from CoP movement were sampled at 1000-Hz and filtered (0.04–10 Hz band-pass, Butterworth zero-lag fourth order). Analysis was performed with Kistler MARS software (MARS 5.0, Kistler Instruments AG, Winterthur, Switzerland). The average velocity of CoP movement in the anterior–posterior (AP) direction and the mean frequency of changes in CoP movement during AP postural sway were used for further analysis of CoP movement during balance tests as suggested by previous research [34].

The accuracy of head and neck movements during the Butterfly test was analyzed using the NeckSmart software applying the following parameters: mean and standard deviation of the relative time spent on the target during each trial expressed as a percentage of total trial time (time-on-target), relative time spent behind the target (undershoot), and time spent in front of the target (overshoot). Averages and standard deviations for all conditions were used for further analysis.

Accuracy of the head-to-neutral relocation, representing position sense in angular degrees (°) was analyzed in NeckSmart software. The following parameters were calculated: mean of the absolute deviation from the neutral position over the three trials for each measured direction (absolute error), average magnitude of both under and overestimation of target position (constant error) and variability of three consecutive repetitions expressed as two standard deviations (variable error).

The procedure of analyzing eye movement data is described in the study by Majcen Rosker et al. [6]. The square waves, saccades and blinks were removed from the eye movement data using custom-written software in Matlab (R2017b, MathWorks, Natick, MA, USA). The sixth to ninth cycles were used for further analysis. Horizontal eye movements were analyzed using gain, calculated as the ratio between eye velocity amplitude and visual target velocity amplitude as described by Tjell et al. [21] during right (gain R) and left (gain L) neck torsion, as well as the difference between gain at neck torsion and neutral position (SPNTdiff).

### 2.4. Statistical Analysis

All statistical analysis was performed in Statistical analysis software (SPSS 23.0 software, SPSS Inc., Chicago, IL, USA). The normality of distribution was analyzed for all independent variables (parameters of the Butterfly and head-to-neutral tests) using skewness and kurtosis and homogeneity of variance using the Lavine’s test. Multiple regression with a best fit model was used to analyze relations between dependent variables (individual balance or SPNT test parameter) and independent variables. Multiple regression was performed in two steps. First, clusters of three difficulty levels (easy, medium and difficult) or three precision measures (absolute error, constant error and variable error) for individual parameters of kinesthetic tests were used for linear model building. In the second step, difficulty levels or precision measures presented in statistically significant models for all variables used to assess relationship to one dependent variable were combined to build the final model. The latter was used to describe the relationship between independent variables (for each kinesthetic test separately) and one dependent variable. Before building each model, the level of collinearity was analyzed. For each model, adjusted R2 and *p* values were calculated (treated as significant when *p* < 0.05).

## 3. Results

### 3.1. Participants Characteristics

Eighty-five individuals participated in the study, 43 idiopathic neck pain patients and 42 asymptomatic controls (demographic data and results of magnetic imaging assessment are presented in Table 1).

### 3.2. Multiple Regression Analysis for Butterfly Test and Postural Balance

Results of the multiple regression analysis between the Butterfly tests and individual dependent variables (balance Vap or Fap) are presented in Table 2 Only statistically significantly models were presented.

Statistically significant models produced by the first step multiple regression modelling presenting a relationship between the body sway parameters (Vap or Fap) and the butterfly test parameters are presented in Table 1. Vap when performing balance in neutral position proved to have no relation with the Butterfly test in either of the groups. For neck torsion position, the patient group presented with relations to the butterfly test. Such relationships were only observed for the left neck torsion in the control group. Fap proved to have some relations to the Butterfly test parameters in the neutral position in both groups. In the neck torsion position, the patients presented with statistically significant relations when observing the right neck torsion and patients when observing the left neck torsion. In general, all above-described relations tended to be more pronounced in neck pain patients than in healthy controls.

Second level multiple regression was unable to upgrade the models presented at the first level of multiple regression in the neutral position. Under left neck torsion, the multiple regression presented with a superior Vap variability, describing the model using a time-on-target standard deviation at the difficult level and an overshoot standard deviation at the medium level (R^2^ = 0.452; F = 6.247; 0.012) in neck pain patients. In the healthy group, no such model was observed for Vap, as no improvement was observed by combining time-on-target standard deviation and overshoot standard deviation at the medium difficulty. At right neck torsion no superior models to the first level multiple regression could be observed for both groups. No collinearity between variables was observed.

### 3.3. Multiple Regression Analysis for Head-to-Neutral Position Sense and Postural Balance

Results of multiple regression analysis between head-to-neutral position sense and individual dependent variables (balance Vap or Fap) are presented in Table 3. Only statistically significantly models are presented.

Both, patient and control presented with some relations between the head-to-neutral test and the Vap or Fap in the neutral stance. The patient group presented with some but weak relations between the head-to-neutral test and Vap under both neck torsion positions. No second level multiple regression models were built due to only one parameter being significant at the first level for all neck positions in both groups. No collinearities were observed.

### 3.4. Multiple Regression Analysis for Butterfly and SPNT Test

Results of multiple regression analysis between Butterfly parameters and individual dependent variables (Gain at different neck positions or SPNTdiff) are presented in Table 4. Only statistically significantly models were presented.

Both patients and the control group presented with some relations between the Butterfly parameters and gain at the neutral position. Such relations under neck torsion position were only observed for the patient group in both neck torsion positions. The SPNTdiff only presented with the relation to the overshoot standard deviation in the patient group. No second level multiple regression models could be built to present superior characteristics as observed at the first level. No collinearity was observed.

### 3.5. Multiple Regression Analysis for Head-to-Neutral Relocation Test and SPNT Test

Results of the multiple regression analysis between head-to-neutral relocation test parameters and individual dependent variables (gain at different neck positions or SPNTdiff) are presented in Table 5. Only statistically significantly models are presented.

Only the patient group presented with relations between the head-to-neutral relocation test parameters and gain at neutral and neck torsioned position. The control group presented with low relations between right absolute error and SPNTdiff. No superior models were observed at the second level multiple regression model. No collinearity was observed.

## 4. Discussion

The aim of this study was to investigate the relationship between cervicocephalic kinesthesia using the Butterfly test and the head-to-neutral paradigm and balance under neck torsion positions or oculomotor control during SPNTT in idiopathic neck pain patients and healthy controls. Results from our study confirm a relationship between kinesthetic awareness of the neck measured with both tests and postural balance in neck pain patients, which is less prominent in healthy individuals. A relationship between cervicocephalic kinesthetic awareness and gain during neck torsion maneuver, with less SPNTdiff, was observed in idiopathic neck pain patients, and a smaller relationship was found in healthy individuals.

While cervicocephalic position sense and postural balance have been extensively studied separately in patients with neck pain disorders [35] their relation has been seldom investigated [3]. In the research by Treleaven et al. [3] the relationship between balance and the head-to-neutral relocation test presented with small to medium correlations. Our study importantly upgrades their findings where the relationship between neck position sense and balance was more pronounced in neck torsion maneuvers. This is more evident in neck pain patients and less so in healthy individuals. Assessing balance tasks when the body is rotated underneath the stationary head is thought to stimulate cervical but not vestibular receptors. In patients with neck pain disorders, abnormal cervical afferents can contribute more to sensorimotor disturbances when the neck is in a torsioned position [36]. A previously proposed mechanism of cervical-driven postural balance deficits can therefore be confirmed by the more pronounced relationship between neck kinesthesia and balance in neck torsion position found in our study, which was evident in both observed groups.

Previous studies assessing neck pain patients found a poor ability to control body sway in the AP direction [34]. Our results presented with a medium relationship between average CoP movement velocity and frequency in the AP direction and some of the Butterfly parameters (time-on-target standard deviation, time-on-target and overshoot). The altered inter-trial standard deviation of the Butterfly test parameters could indicate less efficient sensorimotor control of cervical spine movements, which is in line with other studies confirming alterations in head movement variability in patients with neck pain disorders [37]. The altered variability of head and neck movements found during the Butterfly test in our study is related to body sway velocity and frequency in the AP direction. This is partially in line with other studies, where cervical spine injury was associated with altered body stiffness in postural and locomotion tasks [37,38]. In general, postural and locomotor tasks are dependent on a closed-loop mechanism, where movement corrections are based on proprioceptive feedback [39]; however in neck pain patients, this closed-loop system could be hampered due to commonly observed sensory mismatch. Another parameter that was related to body sway control was the overshoot of the head and neck movements while tracking an unpredictable moving target. The largest proportion of overshoot can be caused by a less efficient correction of movement direction. In this instance, it is necessary to accurately reverse the function of the muscles performing the initial impulse from agonists to antagonists in order to accurately decelerate and initiate changes in movement direction. Accurate movement control demands appropriate sensory feedback [40]; therefore, proprioceptive deficits could result in inappropriate sensory motor coupling, consequently affecting balance.

It could be speculated that consistency of the time spent on target in the Butterfly test could be dependent on the variation in adjusting for the cervical kinesthetic perception error, which is an important mechanism in postural control. One of the possible drivers of perception error, besides the pathological ingrowth of proprioceptors into the vertebral disc [16], can be increased tonic activity and altered proprioceptive feedback from dorsal cervical muscles commonly seen in patients with neck pain disorders. This could alter their body sway control, which has been suggested by Pettorossi and Schieppati [10], where proprioceptive alterations induced by vibration of dorsal neck muscles significantly influenced body posture and movement in AP direction.

Neck torsion maneuvers have been thought to affect eye movement control due to disturbed proprioceptive feedback from the cervical spine [41] but the relationship between cervical kinesthesia and oculomotor control has not been thoroughly studied. Treleaven et al. [3] found no correlations between cervical proprioception and eye movement control using only SPNTdiff. Our study in addition to SPNTdiff also analyaed gain. The parameter of gain showed a greater relationship with the head-to-neutral position than SPNTdiff. This is somehow expected, as the gain measured during neck torsion position directly reflects alterations in proprioceptive feedback. SPNTdiff on the other hand reflects the magnitude of disturbances in cervico-ocular and cervico-colic reflexes. To upgrade the current knowledge, the Butterfly test that measures the dynamic head and neck movement control was performed. The results presented a medium relationship with gain but not with SPNTdiff in neck pain patients, but this was less pronounced in asymptomatic individuals. Undershoot and its inter-trial standard deviation were associated with gain performance when neck was torsioned to the left and right. The undershoot parameter could be associated with increased tonic muscular activity via increased muscle spindle firing, which could lead to an inability to react accurately during unpredictable changes in the direction of movement. Increased and asymmetric muscle spindle firing could cause sensory mismatch, consequently influencing oculomotor control via its direct neurophysiological connection to the vestibular and visual system [7,16].

One of the important limitations of our study was a small sample size, which could have affected the level of observed relationships and consequently the models build by the multiple regression method. As patients with neck pain disorders represent a heterogenous group which differs in the type of sensorimotor disturbances, future studies should subgroup them according to the location of pain [16] and the presence of other symptoms such as dizziness and visual disturbances [42].

## 5. Conclusions

It can be concluded that the specific aspect of neck kinesthetic functions (i.e., movement sense but less position sense) importantly contributes towards oculomotor and balance control, which is more evident under a neck torsion position in neck pain patients, but is less pronounced in asymptomatic individuals.

## Figures and Tables

**Table 1:** Demographic data.

	Idiopathic Neck Pain Patients (*n* = 43)	Asymptomatic Controls (*n* = 42)
Gender	31 women/12 men	24 women/18 men
Average age (age range)	41.3 ± 6.7 years (25–51 years)	39.1 ± 6.3 years (23–49 years)
Average pain duration	13.4 ± 9.1 months	
Average VAS score	4.9 ± 1.7	
Disc protrusion or herniation (C4–Th1)	27 patients	
Facet joint osteoarthritis (C5–Th1)	9 patients	
Low grade spondylolisthesis	12 patients	
Cervical spine stenosis	9 patients	
Combination of at least two types of structural deformities	34 patients	
One type of structural deformity	9 patients	

**Table 2:** Relations between body sway and Butterfly test.

Body Sway Parameter	Group	Butterfly Parameter	Difficulty	Neutral			Left			Right		
				R^2^	F	*p*	R^2^	F	*p*	R^2^	F	*p*
**Vap**	**Patients**	**ToTsd**	**D**				0.318	8.472	0.011			
		**Undsd**	**E**				0.477	7.137	0.017			
		**Ovrsd**	**M**				0.423	9.306	0.032			
			**D**							0.054	4.288	0.043
		**Und**	**M**							0.072	8.019	0.023
	**Healthy**	**ToTsd**	**M**				0.216	3.967	0.000			
		**Ovrsd**	**M**				0.360	4.436	0.040			
**Fap**	**Patients**	**ToT**	**M**							0.091	6.783	0.012
		**Und**	**M**							0.089	6.698	0.012
			**D**	0.189	4.738	0.046						
	**Healthy**	**ToTsd**	**M**				0.058	4.588	0.036			
		**ToT**	**M**	0.135	9.047	0.002	0.055	4.363	0.041			
		**Und**	**M**	0.088	6.577	0.013	0.098	4.136	0.021			

R^2^—adjusted R square, F—F statistic, *p*—statistical significance; Neutral—neutral head position; Left—left neck torsion; Right—right neck torsion; Vap—average velocity of CoP movement in anterior-posterior direction; Fap—average frequency of direction change of CoP movement in anterior-posterior direction; ToTsd—time-on-target standard deviation; Undsd—undershoot standard deviation; Ovrsd—overshoot standard deviation; Und—undershoot; ToT—time-on-target; D—difficult trajectory of the Butterfly test; M—medium trajectory of the Butterfly test; E—easy trajectory of the Butterfly test.

**Table 3:** Relations between body sway and head-to-neutral relocation test.

Body Sway Parameter	Group	HTN Direction	HTN	Neutral			Left			Right		
				R^2^	F	*p*	R^2^	F	*p*	R^2^	F	*p*
**Vap**	**Patients**	**Right**	**Ae**	0.066	5.124	0.027				0.208	4.105	0.040
	**Healthy**	**Right**	**Ae**				0.083	3.629	0.033	0.101	7.551	0.038
**Fap**	**Patients**	**Forward**	**Ce**	0.055	4.379	0.041						
		**Right**	**Ae**									

HTN direction—direction of the head-to-neutral relocation test; HTN—head-to-neutral relocation accuracy parameter; R^2^—adjusted R square, F—F statistic, *p*—statistical significance; Neutral—neutral head position; Left—left neck torsion; Right—right neck torsion; Vap—average velocity of CoP movement in anterior-posterior direction; Fap—average frequency of direction change of CoP movement in anterior-posterior direction; Ae—absolute error; Ce—constant error.

**Table 4:** Relations between eye movements during smooth pursuit neck torsion test and the butterfly test.

Group	Butterfly Parameter	Difficulty	Gain_n			Gain_l			Gain_r			SPNTdiff		
			R^2^	F	*p*	R^2^	F	*p*	R^2^	F	*p*	R^2^	F	*p*
**Patients**	**ToTsd**	**E**	0.421	8.278	0.018	0.397	9.025	0.031	0.391	8.316	0.041			
	**Undsd**	**E**				0.372	9.935	0.022	0.423	8.333	0.018			
	**Ovrsd**	**E**	0.529	7.869	0.011							0.293	3.298	0.044
	**ToT**	**E**				0.306	5.418	0.045	0.364	6.729	0.029			
	**Und**	**E**				0.417	8.164	0.019	0.495	10.804	0.029			
**Healthy**	**ToTsd**	**D**	0.287	2.717	0.042									
	**Undsd**	**M**	0.325	2.002	0.034									
	**Over**	**E**							0.265	9.932	0.049			

Gain_n—gain in neutral neck torsion position; Gain_l—gain at left neck torsion position; Gain_r—gain at right neck torsion position; R^2^—adjusted R square, F—F statistic, p—statistical significance; ToTsd—time-on-target standard deviation; Undsd—undershoot standard deviation; Ovrsd—overshoot standard deviation; Und—undershoot; ToT—time-on-target; Over—overshoot; D—difficult difficulty; M—medium difficulty; E—easy difficulty.

**Table 5:** Relations between eye movements during smooth pursuit neck torsion test and the head-to-neutral relocation test.

Group	HTN Direction	HTN	Gain_n			Gain_l			Gain_r			SPNTdiff		
			R^2^	F	*p*	R^2^	F	*p*	R^2^	F	*p*	R^2^	F	*p*
**Patients**	**F**	**Ae**	0.476	5.543	0.031									
	**R**	**Ve**	0.387	7.318	0.024	0.519	11.803	0.007	0.420	8.235	0.018			
	**L**	**Ae**	0.413	8.92	0.039									
**Healthy**	**R**	**Ae**										0.138	13.676	0.048

HTN direction—direction of the head-to-neutral relocation test; HTN—head-to-neutral relocation accuracy parameter; Gain_n—gain in neutral neck torsion position; Gain_l—gain at left neck torsion position; Gain_r—gain at right neck torsion position; R^2^—adjusted R square, F—F statistic, *p*—statistical significance; F—forward head movement during the head-to-neutral test; R—right head movement during the head-to-neutral relocation test; L—left head movement during the head-to-neutral relocation test; Ae—absolute error; Ve—variable error.

## Data Availability

The data presented in this study are available on request from the corresponding author.
